# OP63 Life Cycle Health Technology Assessment: Brazil’s Experience With CONITEC

**DOI:** 10.1017/S0266462325101207

**Published:** 2025-12-29

**Authors:** Lais Lessa Neiva Pantuzza, Ana Carolina de Freitas Lopes, Barbara Pozzi Ottavio, Luciene Fontes Schluckebier Bonan, Amanda Oliveira Lyrio

## Abstract

**Introduction:**

Life cycle health technology assessment (LC-HTA) refers to continuous methodologies used to evaluate, reassess, and de-adopt technologies. In LC-HTA, the consequences of using a technology are assessed throughout its life cycle, from development to obsolescence. This study examined the experience of the National Committee for Health Technology Incorporation (CONITEC), the Brazilian Health Technology Agency, in applying LC-HTA at various stages of the health technology life cycle.

**Methods:**

This study analyzed CONITEC’s implementation of the LC-HTA by reviewing publicly available recommendation reports and other data published by the Committee. A systematic analysis of these documents was conducted to identify and categorize activities performed across the health technology life cycle: pre-commercialization, post-commercialization, and disinvestment. The analysis aimed to identify when each LC-HTA activity was first implemented and to understand how these processes are operationalized within the Brazilian public health system.

**Results:**

CONITEC has implemented LC-HTA activities across various life cycle stages, including pre-commercialization, post-commercialization, and disinvestment. Since 2014, early awareness syntheses have evaluated technologies with potential health system impacts or emerging technologies for priority diseases. Horizon scanning identifies new and emerging technologies that could compete with the technology under evaluation by CONITEC and has been included in every report since 2017. Implementation monitoring, consistently conducted since 2020, prioritizes technologies with uncertainties regarding safety, effectiveness, or economic outcomes. Disinvestment activities, initiated in 2013, target technologies no longer used in the Brazilian public healthcare system, optimizing resources and improving decision-making.

**Conclusions:**

LC-HTA activities were independently implemented by CONITEC, but not all health technologies are routinely evaluated across all phases of their life cycle. LC-HTA implementation is most beneficial in specific contexts, such as during a public health crisis (e.g., a pandemic) or for technologies deemed strategically important for the country. However, each phase-specific activity has yielded valuable results, contributing to evidence-based decision-making.

